# In-Line Wood Defect Detection Using Simple Scalar Network Analyzer

**DOI:** 10.3390/s22239495

**Published:** 2022-12-05

**Authors:** Mohamed Radwan, Noah Becker, David V. Thiel, Hugo G. Espinosa

**Affiliations:** School of Engineering and Built Environment, Griffith University, Brisbane, QLD 4111, Australia

**Keywords:** timber, scalar network analyzer, microwave, nearfield scanning, sawmill automation

## Abstract

Timber is widely used in new structures. The leading causes of structural failure are sited at bolt connections, cavities, and knots. This paper introduces a simple method to detect bolts in wood using a UHF Scalar Network Analyzer (SNA). The electronics placed inside an aluminum box with a slot aperture transmit a microwave signal through the slot, and the near-field signal determines the reflection coefficient (S_11_). Major changes from baseline are an accurate method to detect cavities and bolts inside the wood. Experiments were conducted on pinewood beams with cross-section dimensions of (70 mm × 70 mm). The scalar network analyzer circuit can detect bolts and cavities within a 30 mm range from the wood surface. The technique can be used for timber beam preparation in an automated sawmill at speed.

## 1. Introduction

Population growth and urbanization worldwide require the increased production of cement, steel, and other building materials. These materials are a significant source of greenhouse emissions. Engineers have a role in encouraging less carbon-intensive production methods and maintaining sustainability simultaneously. Wood is a natural, sustainable building material with a bio-original long-term lifespan and so represents a very desirable alternative construction material [1,2,3,4].

The use of wood products is a means to mitigate climate change. Using wood can reduce the energy usage and carbon emissions associated with the construction and demolition phases of building life cycles. In [5], a comparative study of two equivalent buildings constructed with a wooden frame and a reinforced concrete frame revealed that the manufacture of wooden-constructed buildings uses 28% less energy and emits 45% less carbon than the manufacture of materials for concrete buildings. In [6], a similar study shows that a 17% increase in wood usage in the New Zealand building industry could result in a 20% reduction in carbon emissions from the manufacture of all building materials, and 1.5% of New Zealand’s total emissions. In [7], substituting conventional building materials (i.e., concrete and steel) for mass timber, reduces construction phase emissions by 69%, and could provide as much as 9% of the global emissions reduction needed to meet 2030 targets for keeping global warming below 1.5 °C.

There is increasing worldwide demand for lumber [8], and several new non-destructive testing (NDT) methods based on different physical principles are now in everyday use [9,10]. In [11], NDT methods are the main tool for effective waste management in the construction process. Mechanical and physical features of wooden buildings can be assessed using different NDT methods such as ultrasonic, radiographic, and trimming resistance test methods. In addition, internal defects of the wooden structure can be assessed using macroscopic evaluation, acoustic, and radiographic methods. In general, the NDT of wood can help in treating waste wood products to be raw materials during the construction and demolition waste management process.

The demand for the optimal evaluation of raw wood material through detection and classification into quality classes resulted in semi-destructive and non-destructive methods for wood properties assessment. Resistograph is a semi-destructive method that partially damages the wood log or pole without affecting its further use [12]. Acoustic and ultrasonic methods use sound and ultrasonic waves to identify wood properties. The disadvantages of using the previous methods are that they are difficult to apply, and they require fast acoustic tools to evaluate wood beams during manufacture [11]; they also require an additional destructive laboratory test for precise results [12].

A study to nondestructively detect defects such as knots and grain deviation using a flexible vibration method is introduced in [13]. Essentially, the theoretical wave shape is compared with the wave shapes obtained when tapping wooden beams.

Electromagnetic methods of investigation include microwave tomography and can yield information about moisture, density, fiber direction, and the location of defects. Unfortunately, this technique requires access to all sides of the wood sample and broadband vector network analyzer scanning [14].

A study to classify solid wood flooring based on its features to meet the artistic effects and meet the individual needs of customers was introduced in [15]. Charged-coupled device (CCD) cameras were used to collect solid wood floor images and then classify them using machine vision, deep learning methods, and ensemble learning methods. In [16], machine vision technology and unsupervised learning models were used to classify images collected by a CCD camera to classify wood floor colors and improve the appearance of wood products assembled from multiple panels. The previous methods have high accuracy in classifying wood beam colors using machine learning and do not provide a solution for hidden anomalies beneath the wood surface.

We recently introduced a near-field, microwave scanning technique using a cavity-backed slot antenna [17] using 4.4 GHz in both reflection and transmission modes. With access to a single planar face, the structural health monitoring of intact timber structures is also of significant interest [18,19,20]. In this paper, we report additional refinements to this technique with the following aims:

- To reduce the microwave frequency for increased depth of penetration.
- To reduce the equipment costs through the construction of a single-frequency scalar network analyzer (SNA).
- To demonstrate the system sensitivity by scanning known subsurface defects in timber.

## 2. Materials and Methods

The resonant frequency of a cavity-backed slot antenna depends on the size of the cavity, the location of the feed element, the size of the slot, and the relative permittivity of the wood. In order to reduce external interference from Wi-Fi and Bluetooth devices, the selected frequency of operation was 2.3 GHz.

**Figure 1:**
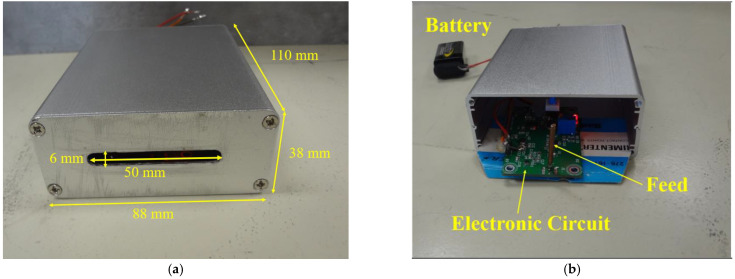
The SNA cavity-based slot antenna with internal electronics: (**a**) The front face of the SNA assembly showing the aluminum cover with the radiating slot; (**b**) The electronic circuit with the vertical antenna feed is shown at the front of the box but was located inside the aluminum box for measurements.

**Figure 2:**
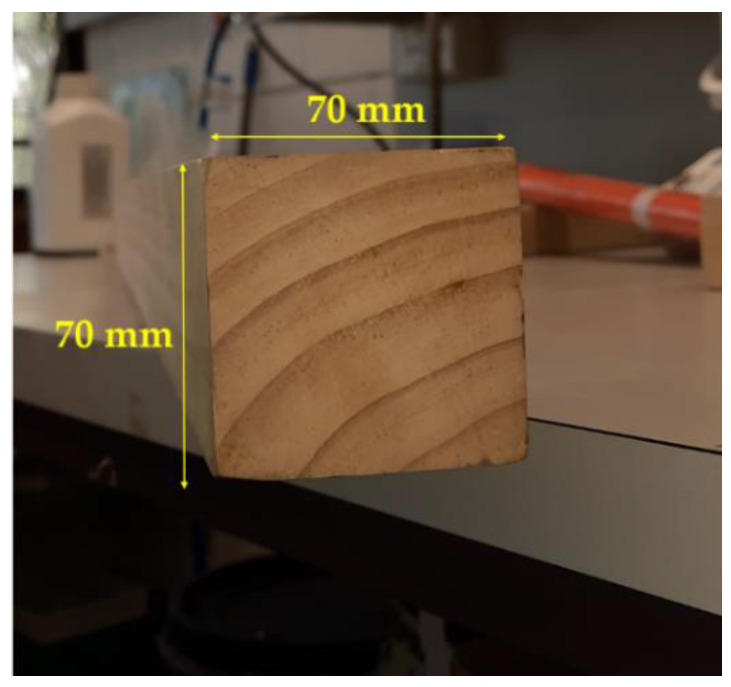
Pinewood beam (cross-section dimensions: 70 mm × 70 mm).

A rectangular aluminum box was used for the reflection measurements (internal dimensions: 110 mm × 88 mm × 38 mm) with an open resonant slot (6 mm × 50 mm) as shown in Figure 1a. The electronic circuit for the SNA was positioned inside the box (Figure 1b) and clamped to the vertical face of a wooden mitre box. External output DC connections (power and sensor outputs) were connected to a digital storage oscilloscope. The wood beam under investigation was moved step-wise across the front of the slot. The pinewood beam (cross-section dimensions: 70 mm × 70 mm) was sourced from a local timber merchant (Brisbane, QLD, Australia) and commercially prepared (kiln-dried with a moisture content of 17%) and smoothed for all four sides (Figure 2). The electric field from the SNA is linearly polarized with the electric field perpendicular to the long axis of the slot. The SNA will therefore be most sensitive to defects and imperfections in the wood with an orientation that is parallel to the electric field.

**Figure 3:**
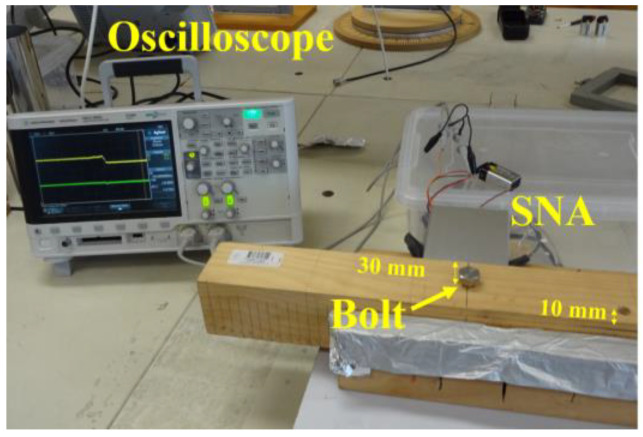
Experiment setup showing the timber under test containing a metallic bolt at 30 mm from the vertical surface and a 10 mm hole from the opposite vertical surface. The DC connections outside the box included the output lines and the DC power supply. The DC output displayed on the oscilloscope shows the change when the bolt was inserted into the wood.

The internal feed from the oscillator circuit was a vertical brass monopole (shown in Figure 1b). The input impedance of the complete circuit is sensitive to the material covering the antenna slot. Figure 3 shows the experimental arrangement with the pinewood sample located against the SNA slot. The DC output from the detector circuit was displayed on an oscilloscope.

The electromagnetic wave propagates through the air-wood boundary with different electromagnetic impedance. The antenna impedance reflection coefficient (*S*_11_) can then be defined as:

(1) $$S_{11}={\left(\frac{P_{ref}}{P_{inc}}\right)}^{1/2}\propto \;\sqrt{V_{ref}}$$

where *P_ref_* and *P_inc_* are the reflected and incident power levels from the antenna, respectively, and *V_ref_* is the measured voltage.

## 3. SNA Detection Circuit

A voltage-controlled oscillator (VCO) (MAX2751) was selected as a signal generator [21]. It generates a UHF frequency signal between 2.4 GHz and 2.5 GHz determined by the input control voltage. The generated signal passes through the directional coupler to the antenna. The reflected signals are received at the isolation port of the coupler. The coupler chosen was the model BDCN-7-25+ to fit the VCO frequency band (0.824 to 2.525 GHz) [22].

**Figure 4:**
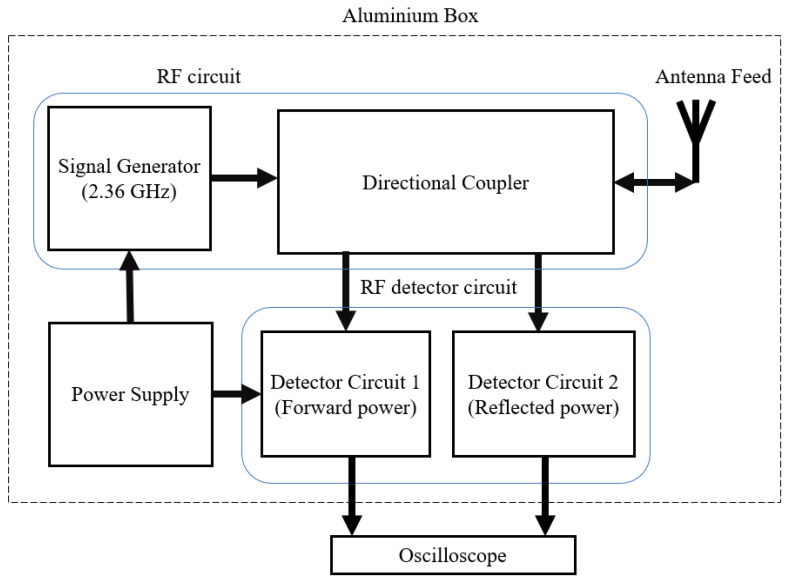
Scalar vector network analyzer block diagram.

The scalar network analyzer (Figure 4) has two detector circuits: detector circuit 1 (MAX2204 RF Power Detector-Maxim Integrated) for the incident power, and circuit 2 (MAX4003 45dB RF Detector-Maxim Integrated) for the reflected power. A four-port directional coupler (BDCA-7-25 high-power bi-directional coupler–minicircuits) was used to separate the transmitted power and the reflected power levels. The detected power levels are converted to DC levels and recorded by an oscilloscope sweep as *V_ref_*. The step level change (Figure 3) corresponds to the time of the insertion of the metallic bolt. The voltage is proportional to the power of the reflected signal given in Equation (1) and so is a measure of the *S*_11_.

The incident power at 2.36 GHz from the signal generator (MAX2751) was found to be constant at 3 dBm for all measurement surfaces undertaken. A low-power detector records the reflected power level (*P_ref_*). The voltage response (*V_ref_*) is approximately linear with the input power measured in dBm with a slope of 0.03 V/dBm over the output voltage range of 0.45–1.5 V [21]. Thus, the variations in *V_ref_* are directly related to the reflected power from the antenna mismatch to the timber. The detector is sensitive to changes in the electromagnetic properties of the wood beam in front of the antenna aperture. This effect was explored for a variety of imperfections created in the pinewood beam.

## 4. Results

### 4.1. Device Validation (Air and Wood Measurements)

**Figure 5:**
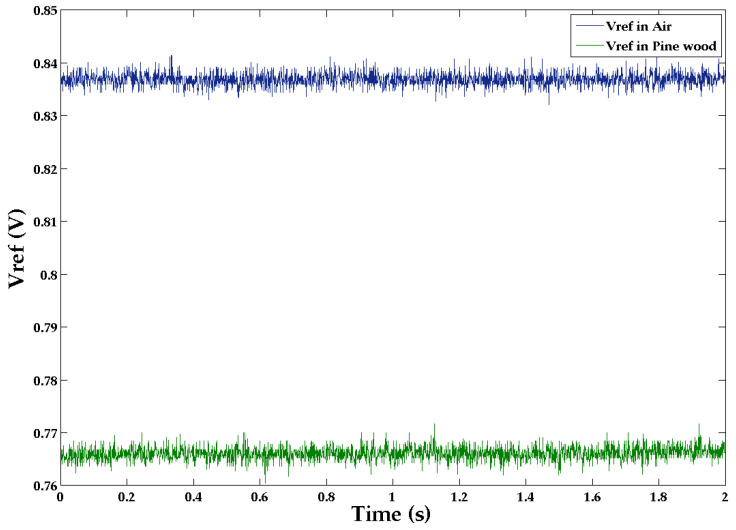
Output voltage *V_ref_* measurements for the antenna slot on both air and pinewood as a function of time.

**Figure 6:**
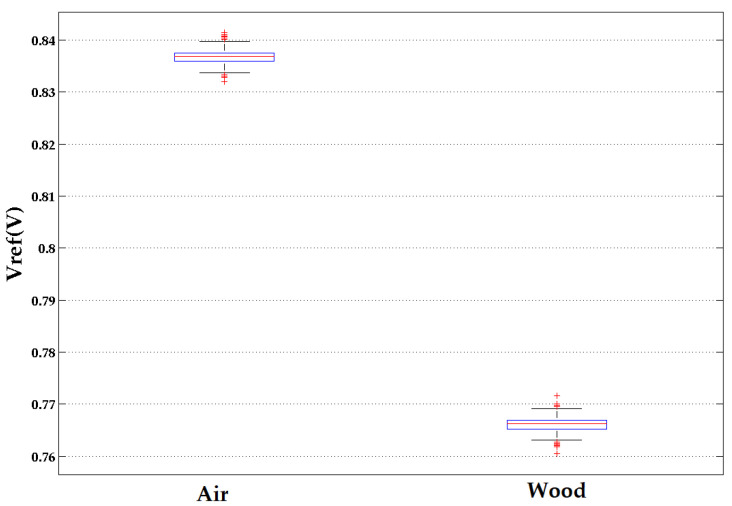
Vref measurements Box and Whisker for air vs. pinewood at a fixed location on the wood at 2.36 GHz. There was a small difference in the noise levels in these measurements.

The generator plus antenna resonant frequency was 2.36 GHz when placed on the flat surface of the wood beam. The change in the output level *V_ref_* from air to wood is shown in Figure 5 and Figure 6 as a function of time for a fixed position on the wood surface. There is a clear difference in the *V_ref_* values.

### 4.2. Steel Bolt in a Wood

**Figure 7:**
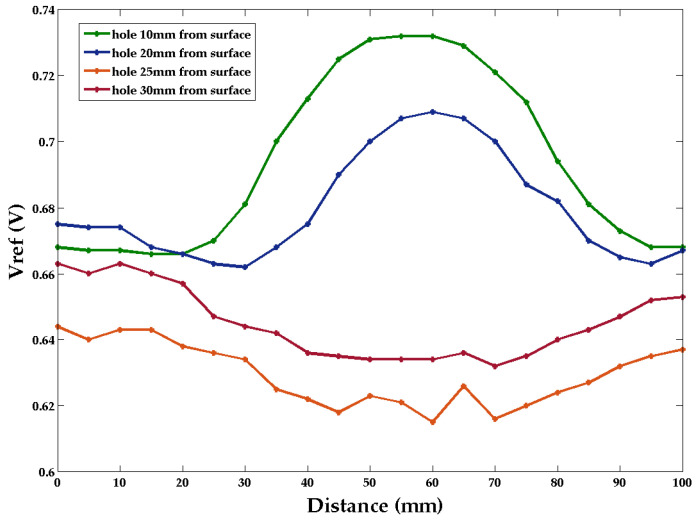
Scan results for bolt in holes 10 mm, 20 mm, 25 mm, and 30 mm from the measurement surface.

Four holes were drilled at different distances from the measurement surface (10 mm, 20 mm, 25 mm, and 30 mm). The wood beam was slowly moved across the SNA touching the wood surface over a 100 mm scanning distance (See Figure 3). With a steel bolt in the holes, the reflected signal voltage was measured as a function of distance (see Figure 7).

With the bolt close to the surface, the deviation is positive (the reflected signal is increased); however, for distances greater than 20 mm, the reflected voltage is decreased (see Figure 7a). The change in *V_ref_* from positive to negative is thought to be the result of a phase reversal in the signal due to the increased separation distance. The deviation in *V_ref_* decreases with increasing distance. Table 1 shows the *V_ref_* values at a 60 mm distance from the start of the scanning position.

### 4.3. Knot Measurements

**Figure 8:**
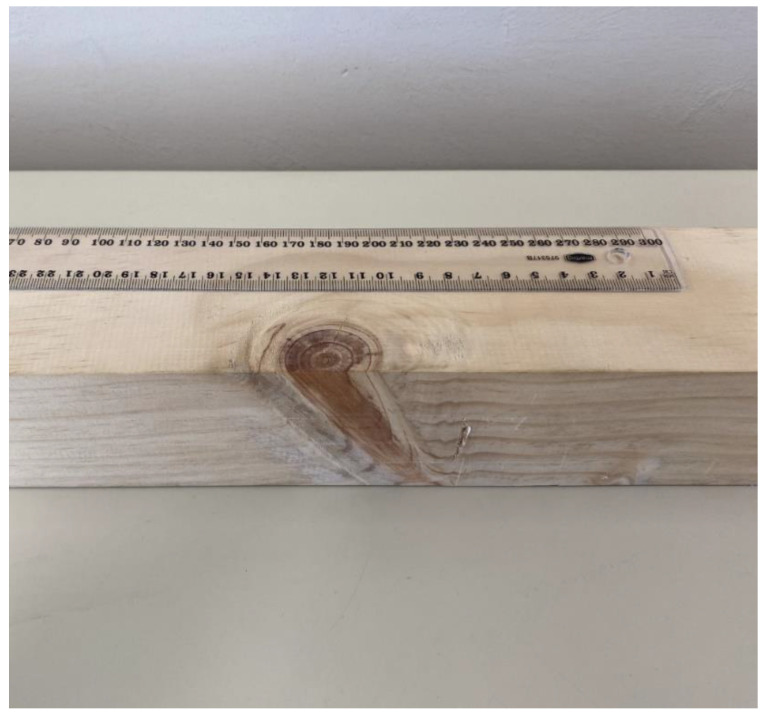
Surface expression of a knot on the pinewood surface.

**Figure 9:**
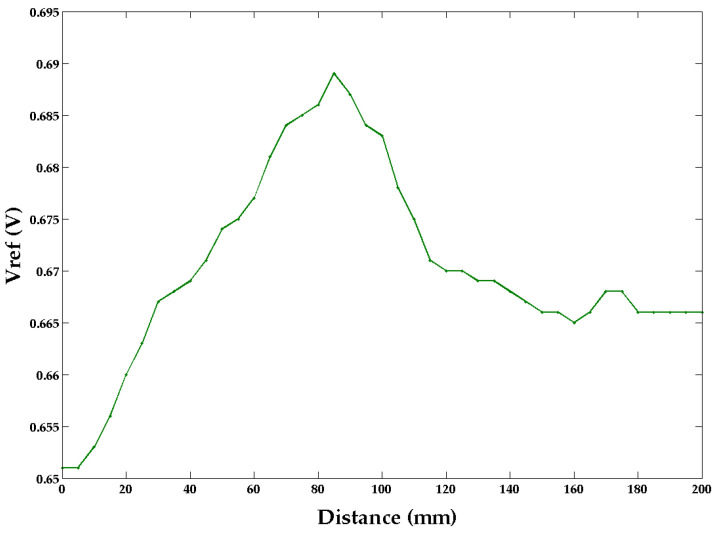
Scan result for the knot on pinewood. The profile is asymmetric as the knot is not perpendicular to the wood grain.

The reflection profile across a large knot (approximately 20 mm in diameter) in the timber (see Figure 8) shows a significant change (Figure 9). As the knot lies at an angle to the perpendicular, the profile is asymmetric, with the left side showing a more abrupt change.

## 5. Discussion and Conclusions

Wood is widely used in new modern structures. Wooden structures can play a role in achieving the United Nations (UN) Sustainability Development Goals (SDGs) [23]. It can contribute to achieving Goal 1: end poverty in all its forms everywhere, and Goal 9: industry, innovation, and infrastructure—build resilient infrastructure, promote sustainable industrialization, and foster innovation. Exploiting local building materials, such as wood, will enhance the creation of resilient, durable, low-cost, and environmentally sound infrastructure.

The simple, battery-powered SNA circuit located in a cavity-backed slot antenna and tuned to 2.36 GHz, has been shown to successfully detect changes in the electromagnetic characteristics of the pinewood. In particular, the circuit was successful in detecting steel bolts at a range of depths from the surface and a major knot in the timber.

The system described can be applied to monitor wood electromagnetic properties either using the hand-held instrument placed on a flat face of a timber beam, or for the automatic characterization of wood during a sawmill operation. There are significant advantages over existing non-destructive testing systems.

Future research directions include the effect of rough wooden surfaces and the effectiveness of the technique in detecting lateral cracks on sawn lumber before and after kiln drying.

## Figures and Tables

**Table 1:** Scan results at 60 mm from the start of scanning.

Hole	*V_ref_* (mV)
10 mm from surface	0.732
20 mm from surface	0.709
25 mm from surface	0.615
30 mm from surface	0.634
